# Mixed-location cerebral microbleeds as a biomarker of neurodegeneration in a memory clinic population

**DOI:** 10.18632/aging.102478

**Published:** 2019-11-25

**Authors:** Bibek Gyanwali, Muhammad Amin Shaik, Chuen Seng Tan, Henri Vrooman, Narayanaswamy Venketasubramanian, Christopher Chen, Saima Hilal

**Affiliations:** 1 Memory Aging and Cognition Centre, National University Health System, Singapore; 2 Department of Pharmacology, National University of Singapore, Singapore; 3 Ageing Research Institute for Society and Education, Nanyang Technological University, Singapore; 4 Saw Swee Hock School of Public Health, National University of Singapore, Singapore; 5 Departments of Radiology and Medical Informatics, Erasmus University Medical Center, Rotterdam, The Netherlands; 6 Raffles Neuroscience Centre, Raffles Hospital, Singapore; 7 Departments of Epidemiology and Radiology and Nuclear Medicine, Erasmus University Medical Center, Rotterdam, The Netherlands

**Keywords:** mixed-location microbleeds, neurodegeneration, hypertensive arteriopathy, memory clinic

## Abstract

Cerebral microbleeds (CMBs) in the lobar and deep locations are associated with two distinct pathologies: cerebral amyloid angiopathy and hypertensive arteriopathy. However, the role of mixed-location CMBs in neurodegeneration remains unexplored. We investigated the associations between strictly lobar, strictly deep and mixed-location CMBs with markers of neurodegeneration. This study recruited 477 patients from a memory clinic who underwent 3T MRI scans. CMBs were categorized into strictly lobar, strictly deep and mixed-location. Cortical thickness, white matter volume and subcortical structural volumes were quantified using Free-Surfer. Linear regression models were performed to assess the association between CMBs and cerebral atrophy, and the mean difference (β) and 95% confidence intervals (CIs) were reported. In the regression analyses, mixed-location CMBs were associated with smaller cortical thickness of limbic region [β= -0.01; 95% CI= -0.02, -0.00, p=0.007) as well as with smaller accumbens volume [β= -0.01; 95% CI= -0.02, -0.00, p=0.004) and presubiculum region of hippocampus [β= -0.01; 95% CI= -0.02, -0.00, p=0.002). Strictly lobar CMBs were associated with smaller total white matter volume [β= -0.03; 95% CI= -0.04, -0.01, p<0.001] and with region specific white matter volumes. The underlying mechanism requires further research and may involve shared mechanisms of vascular dysfunction and neurodegeneration.

## INTRODUCTION

Cerebral microbleeds (CMBs) are defined as focal round hypointense lesions, 2 to 10 mm in diameter with a blooming effect on T2*weighted/susceptibility weighted scans [1, 2]. CMBs are the consequence of two main pathological processes involving small vessels of the brain: 1) cerebral amyloid angiopathy (CAA) which is characterized by the deposition of amyloid in cortical blood vessels exclusively in lobar regions and, 2) hypertensive arteriopathy, characterized by lipohyalinosis and arteriosclerosis of cerebral blood vessels in deep regions of the brain (basal ganglia, thalamus, and infratentorial) [1]. The region-specific distribution of CMBs suggests the possibility of different underlying etiologies and different risk factors for lobar and deep CMBs [1–3].

CMBs are frequently observed in elderly population with prevalence ranging from 5–28% [4] and are associated with cognitive impairment, functional decline and dementia [1]. Furthermore, co-existence of CMBs with other cerebral small vessel disease markers (SVD) such as WMH, lacunes and cerebral atrophy is common in elderly [4, 5]. Previous studies have shown vascular risk factors such as hypertension, diabetes, hyperlipidemia and increasing age to be linked with brain atrophy [6, 7] and these risk factors are also associated with CMBs [8–10]. Although CMBs and brain atrophy mostly share common risk factors and occur concomitantly, the association between them has not been explored in the Asian population.

It is further reported that lobar CMBs may alter cerebral white matter perfusion leading to brain volume reduction [11, 12]. However, previous studies have shown conflicting results. In CAA subjects, cortical thinning and decreased hippocampus volumes were observed compared to healthy controls but there was no independent association between the number of lobar CMBs and cerebral atrophy [12]. Another study in patients with Alzheimer’s disease (AD) showed an association between lobar CMBs and gray matter atrophy in the temporal lobe [13]. By contrast, one study reported a link between CMBs and higher volumes of basal ganglia and cerebellum [14]. Further studies are thus needed to understand the role of CMBs in neurodegeneration.

Lately, the increased understanding of the significant overlap between vascular and neurodegenerative pathologies have challenged the traditional link of lobar CMBs to AD and deep CMBs to vascular cognitive impairment [15, 16]. With increased recognition of mixed-location CMBs as a common pattern observed in clinical practice [3], recent data has suggested that hypertensive arteriopathy may also cause lobar CMBs [17–19]. Keeping in view the possible synergistic or additive effects of hypertensive arteriopathy on CAA, mixed-location CMBs may potentially play a key role in both gray and white matter damage [3]. So far, previous studies were mainly focused on lobar and deep CMBs and ignored the most common type of CMBs i.e. mixed-location CMBs. Furthermore, these studies did not examine the associations with cortical, subcortical and white matter atrophy in detail according to the location and burden of CMBs. Finally, none of these studies were from a memory clinic population with mixed pathology.

The present study aims to investigate the associations between strictly lobar, strictly deep and mixed-location CMBs with markers of neurodegeneration including gray matter (cortical thickness, subcortical structural volumes including hippocampal subfields) and white matter volume in an Asian memory clinic population. Furthermore, we aim to explore whether the effect of CMBs distribution (strictly lobar, strictly deep and mixed-location) on neurodegeneration differs between patients with no cognitive impairment (NCI), cognitive impairment no dementia (CIND) and dementia.

## RESULTS

### Characteristics of participants

Table 1 shows the characteristics of the patients with and without CMB. Of 477 patients, 41.9% (n=200) had at least 1 CMB. Among 200 patients, 53.5% (n=107) were strictly lobar, 17.5% (n=35) were strictly deep and 29.0% (n=58) were mixed-location CMBs. Among the patients with mixed-location CMBs (n=58), the median number of lobar CMBs was 5 and the median number of deep CMBs was 2. Patients with CMBs had higher white matter hyperintensities (WMH) volume (p<0.001) and had a higher prevalence of lacunes (p=0.032) compared to those without CMB. There were no differences in demographics, vascular risk factors (hypertension, hyperlipidemia and diabetes), total enlarged perivascular spaces (ePVS) and neurodegenerative markers (global cortical thickness, subcortical, total white matter and total intracranial volume) in patients with and without CMBs (p>0.05).

**Table 1:** Characteristics of patients with and without cerebral microbleeds.

**Characteristics**	**Without CMBs (n=277)**	**With CMBs (n=200)**	**p- value**
Age (years), mean (SD)	72.6 (8.1)	73.8 (7.6)	0.123
Females, n. (%)	163 (58.8)	106 (53.0)	0.204
***Vascular risk factors***			
Hypertension, n. (%)	187 (67.5)	137 (68.5)	0.819
Hyperlipidemia, n. (%)	198 (71.5)	144 (72.0)	0.901
Diabetes mellitus, n. (%)	97 (35.0)	70 (35.0)	0.997
***Cerebrovascular disease markers***			
Presence of lacunes, n (%)	73 (26.4)	71 (35.5)	**0.032**
Total WMH volume (ml), mean (SD) (log transformed)	1.4 (0.9)	1.9 (1.2)	**<0.001**
Total ePVS, median (IQR) (n=328)	16 (11)	17.5 (9.2)	0.319
***Neurodegenerative markers***			
Global cortical thickness (mm), mean (SD)	2.3 (0.1)	2.2 (0.1)	0.052
Subcortical structures volume (ml), mean(SD)	56.4 (8.0)	55.1 (8.0)	0.096
Total white matter volume (ml), mean (SD)	354.6 (61.9)	358.1 (72.5)	0.567
Total intracranial volume (ml), mean (SD)	1113.6 (146.0)	1140.8 (158.6)	0.053

SD, standard deviation; IQR, interquartile range; ml, milliliters; mm, millimeters; CMBs, cerebral microbleeds; ePVS, enlarged perivascular spaces; WMH, white matter hyperintensities.

Bold values represents statistically significant associations at p<0.05.

### Association between CMBs and cortical thickness

Table 2 shows the association between CMBs and cortical thickness. Mixed-location CMBs were associated with smaller global cortical thickness (p<0.005). Region specific analysis showed that mixed-location CMBs were associated with smaller cortical thickness in frontal, temporal and limbic lobes (p<0.05). On applying Bonferroni correction, the association between mixed-location CMBs and cortical thickness of limbic lobe remained significant. On categorizing mixed-location CMBs into presence of 2-4 CMBs and presence of >4CMBs; the presence of >4 CMBs were associated with smaller global cortical thickness as well as region specific cortical thickness in frontal, temporal, limbic and insular lobes when compared to patients with no CMBs. Most of these associations survived multiple testing. Similar associations were observed when CMBs locations (strictly lobar, strictly deep and mixed-location) were treated as categorical data (presence vs absence) [Supplementary Table 1]. No association was observed between strictly lobar and strictly deep CMBs with global and region specific cortical thickness.

**Table 2:** Association between CMBs and cortical thickness.

	**Global cortical thickness mean difference (95%CI)**	**Region specific cortical thickness, mean difference (95%CI)**					
		**Frontal**	**Parietal**	**Temporal**	**Occipital**	**Limbic**	**Insula**
**Strictly lobar CMBs (counts)**	-0.00 (-0.02, 0.02) p=0.983	0.01 (-0.01, 0.03) p=0.406	-0.00 (-0.02, 0.01) p=0.674	-0.00 (-0.02, 0.01) p=0.673	-0.01 (-0.02, 0.01) p=0.582	-0.00 (-0.02, 0.02) p=0.922	0.01 (-0.01, 0.02) p=0.567
**1 CMB**	-0.06 (-0.35, 0.22) p=0.665	0.05 (-0.24, 0.32) p=0.751	-0.17 (-0.45, 0.12) p=0.244	-0.05 (-0.33, 0.29) p=0.8555	-0.11 (-0.39, 0.17) p=0.444	0.13 (-0.15, 0.41) p=0.370	0.17 (-0.21, 0.46) p=0.259
**2-4 CMBs**	-0.03 (-0.35, 0.30) p=0.860	0.11 (-0.22, 0.44) p=0.507	-0.08 (-0.40, 0.24) p=0.628	-0.03 (-0.35, 0.29) p=0.855	-0.14 (-0.46, 0.17) p=0.369	-0.10 (-0.42, 0.23) p=0.562	0.15 (-0.19, 0.48) p=0.384
**>4 CMBs**	0.00 (-0.48, 0.47) p=0.991	0.10 (-0.38, 0.59) p=0.675	-0.07 (-0.54, 0.40) p=0.756	-0.15 (-0.62, 0.32) p=0.527	0.10 (-0.36, 0.56) p=0.671	-0.01 (-0.48, 0.45) p=0.950	0.11 (-0.38, 0.59) p=0.665
**Strictly deep CMBs (counts)**	-0.05 (-0.18, 0.07) p=0.407	-0.08 (-0.21, 0.05) p=0.247	0.02 (-0.10, 0.15) p=0.721	-0.07 (-0.20, 0.05) p=0.262	-0.10 (-0.22, 0.03) p=0.131	-0.04 (-0.16, 0.09) p=0.577	-0.09 (-0.23, 0.00) p=0.155
**1 CMB**	0.14 (-0.26, 0.54) p=0.493	0.11 (-0.30, 0.52) p=0.913	-0.01 (-0.30, 0.50) p=0.615	0.26 (-0.14, 0.65) p=0.199	-0.01 (-0.38, 0.40) p=0.970	-0.03 (-0.37, 0.43) p=0.879	0.26 (-0.15, 0.67) p=0.220
**2-4 CMBs**	-0.25 (-0.86, 0.37) p=0.432	-0.22 (-0.85, 0.40) p=0.484	-0.59 (-1.32, 2.50) p=0.542	-0.25 (-0.85, 0.35) p=0.410	-0.42 (-1.02, 0.17) p=0.165	-0.20 (-0.80, 0.41) p=0.528	-0.34 (-0.97, 0.29) p=0.287
**>4 CMBs**	-0.76 (-2.65, 1.14) p=0.435	-1.21 (-3.16, 0.75) p=0.227	0.60 (-1.29, 2.49) p=0.533	-1.25 (-3.13, 0.63) p=0.193	-1.03 (-2,89, 0.83) p=0.278	-0.27 (-2.17, 1.64) p=0.784	-1.29 (-3.25, 0.68) p=0.199
**Mixed-location CMBs (counts)**	**-0.01 (-0.02, -0.00) p=0.015^#^**	**-0.01 (-0.02, -0.00) p=0.030^#^**	-0.01 (-0.01, 0.00) p=0.136^#^	**-0.01 (-0.02, -0.00) p=0.016**	-0.01 (-0.01, 0.00) p=0.078^#^	-0.01 (-0.02, -0.00) p=0.007*^#^	-0.01 (-0.01, 0.00) p=0.070
**2-4 CMBs**	-0.01 (-0.42. 0.41) p=0.973	-0.01 (-0.42, 0.43) p=0.974	0.13 (-0.28, 0.53) p=0.538	-0.07 (-0.48, 0.34) p=0.725	-0.08 (-0.49, 0.33) p=0.692	-0.06 (-0.48, 0.35) p=0.757	0.11 (-0.32, 0.54) p=0.606
**>4 CMBs**	**-0.42 (-0.76, -0.08) p=0.015^#^**	-0.48 (-0.82, -0.14) p=0.006*^#^	-0.22 (-0.55, -0.11) p=0.189	**-0.43 (-0.76, -0.10) p=0.010**	-0.21 (-0.54, 0.12) p=0.219	-0.54 (-0.87, -0.21) p=0.002*	**-0.45 (-0.80, -0.11) p=0.011**

All values adjusted for age, gender, intracranial volume, hypertension, hyperlipidemia and diabetes.

Bold values represents statistically significant associations at p<0.05.

* Statistically significant after Bonferroni correction (0.05/6 ~ 0.0083).

^#^ Statistically significant after further adjusting for white matter hyperintensities volume, presence of lacunes and total enlarged perivascular spaces (n=328) (p<0.05).

### Association between CMBs and subcortical structures volume

Table 3 shows the associations between CMBs and subcortical structural volumes. Mixed-location CMBs were associated with smaller accumbens volume (p<0.05) and a borderline significance with total hippocampus volume (p=0.075) independent of demographic and vascular risk factors. On applying Bonferroni correction, association with acumbens volume remained significant. Further categorization of mixed-location CMBs into 2-4 and >4 CMBs revealed an association between presence of >4 CMBs with smaller accumbens and brain stem volumes. By contrast, mixed-location CMBs were associated with a larger thalamic volume and presence of >4 mixed-location CMBs were associated with larger lentiform nucleus volume (p<0.05). These associations survived multiple testing. In case of strictly lobar CMBs, 1 CMB and 2-4 CMBs were associated with smaller lentiform nucleus volume and >4 CMBs were associated with smaller hippocampus and brain stem volumes. Most of these associations were still observed in the analysis involving presence and absence of CMBs [Supplementary Table 2]. No significant association was found between strictly deep CMBs with subcortical structures volumes.

**Table 3:** Association between CMBs and subcortical structures volume.

	**Accumbens Mean difference (95%CI)**	**Amygdala Mean difference (95%CI)**	**Lentiform Mean difference (95%CI)**	**Thalamus Mean difference (95%CI)**	**Hippocampus Mean difference (95%CI)**	**Brainstem Mean difference (95%CI)**
**Strictly lobar CMBs (counts)**	-0.00 (-0.02, 0.01) p=0.698	-0.01 (-0.02, 0.01) p=0.433	-0.00 (-0.02, 0.02) p=0.811	-0.01 (-0.03, 0.00) p=0.155	-0.01 (-0.03, 0.00) p=0.070	-0.01 (-0.03, 0.00) p=0.074
**1 CMB**	-0.05 (-0.23, 0.22) p=0.741	-0.11 (-0.36, 0.15) p=0.410	-0.45 (-0.72, -0.18) p=0.001*	-0.09 (-0.33, 0.15) p=0.484	-0.19 (-0.44, 0.07) p=0.155	-0.04 (-0.28, 0.20) p=0.734
**2-4 CMBs**	0.02 (-0.29, 0.32) p=0.924	-0.15 (-0.44, 0.14) p=0.321	**-0.34 (-0.65, -0.03) p=0.030**	-0.06 (-0.33, 0.22) p=0.681	-0.12 (-0.41, 0.17) p=0.415	0.14 (-0.13, 0.41) p=0.315
**>4 CMBs**	-0.13 (-0.58, 0.32) p=0.567	-0.33 (-0.75, 0.09) p=0.123	0.09 (-0.36, 0.53) p=0.699	-0.11 (-0.51, 0.29) p=0.586	**-0.46 (-0.89, 0.04) p=0.032**	-**0.42 (-0.82, -0.02) p=0.039**
**Strictly deep CMBs (counts)**	-0.03 (-0.15, 0.09) p=0.615	0.00 (-0.11, 0.11) p**=**0.985	0.11 (-0.01, 0.23) p= 0.073	-0.00 (-0.11, 0.11) p= 0.995	0.01 (-0.11, 0.12) p=0.882	-0.08 (-0.18, 0.03) p=0.168
**1 CMB**	-0.04 (-0.42, 0.34) p=0.832	0.22 (-0.14, 0.57) p=0.232	-0.02 (-0.40, 0.36) p=0.909	0.21 (-0.12, 0.55) p=0.215	0.25 (-0.11, 0.61) p=0.173	0.01 (-0.33, 0.34) p=0.968
**2-4 CMBs**	0.03 (-0.55, 0.61) p=0.912	-0.15 (-0.69, 0.40) p=0.600	0.17 (-0.41, 0.76) p=0.563	-0.20 (-0.72, 0.31) p=0.439	-0.25 (-0.80, 0.31) p=0.385	**-0.56 (-1.08, -0.04) p=0.034**
**>4 CMBs**	-0.45 (-2.26, 1.37) p=0.629	-0.15 (-1.86, 1.56) p=0.866	1.74 (-0.09, 3.56) p= 0.063	0.00 (-1.62, 1.61) p=0.997	0.23 (-1.50, 1.96) p=0.792	-0.26 (-1.88, 1.36) p=0.753
**Mixed-location CMBs (counts)**	-0.01 (-0.02, -0.00) p=0.004*	-0.00 (-0.01, 0.00) p=0.309	0.00 (-0.00, 0.01) p=0.190	0.01 (0.01, 0.02) p<0.001*	-0.01 (-0.01, 0.00) p=0.075	-0.00 (-0.01, 0.00) p=0.467^#^
**2-4 CMBs**	0.17 (-0.22, 0.56) p=0.383	-0.07 (-0.30, 0.45) p=0.704	0.33 (-0.07, 0.73) p=0.104	-0.16 (-0.51, 0.19) p=0.377	0.14 (-0.24, 0.52) p=0.470	-0.28 (-0.64, 0.07) p=0.113^#^
**>4 CMBs**	-0.60 (-0.92, -0.28) p<0.001*	-0.18 (-0.48, 0.12) p=0.241	0.46 (0.14, 0.78) p=0.005*	-0.25 (-0.54, 0.03) p=0.083	-0.17 (-0.48, 0.14) p=0.281	-0.39 (-0.67, -0.10) p=0.008*

All values adjusted for age, gender, intracranial volume, hypertension, hyperlipidemia and diabetes mellitus.

Bold values represents statistically significant associations at p<0.05.

* Significant after Bonferroni correction (0.05/5 ~0.010).

^#^ Statistically significant after further adjusting for white matter hyperintensities volume, presence of lacunes and total enlarged perivascular spaces (n=328) (p<0.05).

**Figure 1:**
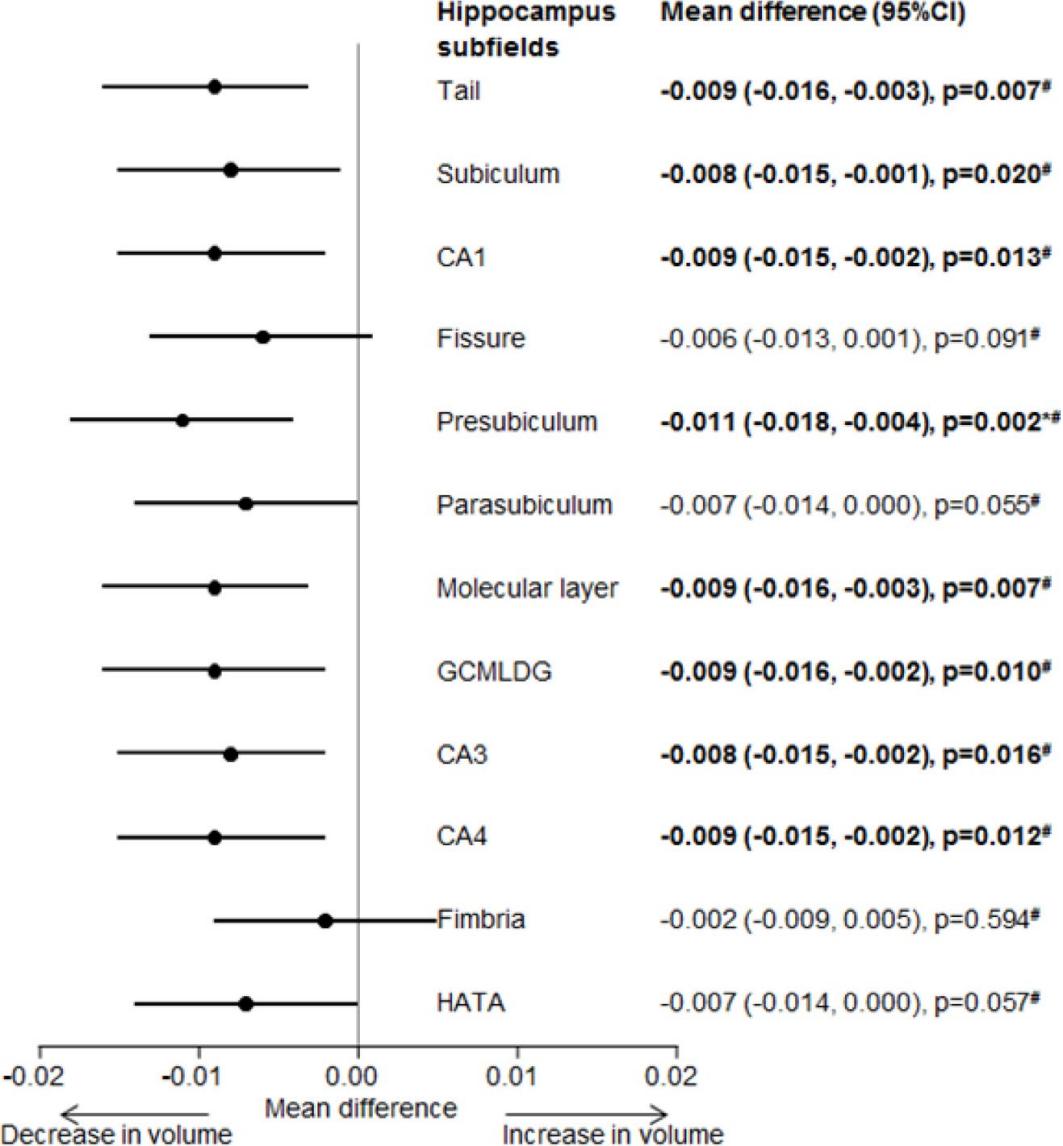
**Forest plot for the association between mixed-location CMBs with hippocampus subfields volume.** Effect estimates adjusted for age, gender, intracranial volume, hypertension, hyperlipidemia and diabetes.Bold values represents statistically significant associations at p =0.05. * Statistically significant after Bonferroni correction (0.05/12 ~ 0.0041). # Statistically significant after further adjusting for white matter hyperintensities volume, presence of lacunes and total enlarged perivascular spaces (n=328) (p<0.05). CMBs, cerebral microbleeds; CA, Cornu Amonis; GCMLDG, Molecular and Granule Cell Layers of the Dentate Gyrus; HAAT, Hippocampal Amygdala Transition Area.

Figure 1 shows the association between mixed-location CMBs and hippocampus subfield volumes. Mixed-location CMBs were associated with smaller volumes of hippocampus tail, subiculum, CA1, presubiculum, molecular layer, GCMLDG, CA3 and CA4 (p<0.05). No significant association was found between strictly lobar and strictly deep CMBs with hippocampus subfield volumes [data not shown]. When treating location of CMBs as categorical data (presence vs absence) no significant association was again found between presence of strictly lobar, strictly deep and mixed-location CMBs with hippocampus subfield volumes [data not shown].

### Association between CMBs and white matter volume

Table 4 shows the association between CMBs and white matter volumes. Strictly lobar CMBs were associated with smaller total white matter volume as well as smaller volumes of frontal, parietal, temporal and occipital white matter (p<0.05). These associations remained significant after applying Bonferroni correction. On categorizing strictly lobar CMBs into presence of 1, 2-4 and >4 strictly lobar CMBs; presence of >4 strictly lobar CMBs were associated with smaller total white matter volume as well as in the frontal, parietal and temporal lobes white matter volumes, which did not survive multiple testing. Deep CMBs were associated with larger parietal, temporal and occipital white matter volume which were stronger in the presence of >4 deep CMBs. On applying Bonferroni correction, only association with occipital lobe remained significant. Similar associations were observed when CMBs locations (strictly lobar, strictly deep and mixed-location) were treated as categorical data (presence vs absence) [Supplementary Table 1].

**Table 4:** Association between CMBs and white matter volume.

	**Total white matter volume (ml) mean difference (95%CI)**	**Lobe specific white matter volume (ml), mean difference (95%CI)**			
		**Frontal**	**Parietal**	**Temporal**	**Occipital**
**Strictly lobar CMBs (counts)**	-0.03 (-0.04, -0.01) p<0.001*^#^	-0.03 (-0.05, -0.01) p<0.001*^#^	-0.02 (-0.04, -0.01) p=0.002*^#^	-0.03 (-0.04, 0.01) p<0.001*^#^	-0.02 (-0.04, -0.00) p=0.010*^#^
**1 CMB**	-0.16 (-0.40, 0.09) p=0.218	-0.13 (-0.38, 0.12) p=0.316	-0.10 (-0.35, 0.15) p=0.448	-0.20 (-0.45, 0.04) p=0.103	-0.16 (-0.41, 0.10) p=0.231
**2-4 CMBs**	-0.09 (-0.37, 0.20) p=0.552	-0.08 (-0.37, 0.20) p=0.573	-0.10 (-0.39, 0.19) p=0.506	-0.08 (-0.36, 0.20) p=0.579	-0.15 (-0.44, 0.15) p=0.329
**>4 CMBs**	**0.45 (-0.86, -0.04) p=0.033^#^**	**-0.42 (-0.83, -0.01) p=0.046^#^**	**-0.45 (-0.87, -0.04) p=0.033^#^**	**-0.47 (-0.87, -0.07) p=0.022^#^**	-0.18 (-0.60, 0.24) p=0.404^#^
**Strictly deep CMBs (counts)**	0.09 (-0.02, 0.20) p=0.0.099	0.01 (-0.11, 0.12) p=0.926	**0.12 (0.01, 0.24) p=0.032**	**0.13 (0.02, 0.24) p=0.016**	0.26 (0.14, 0.37) p<0.001*^#^
**1 CMB**	0.25 (-0.10, 0.59) p=0.166	0.16 (-0.19, 0.51) p=0.370	0.19 (-0.16, 0.54) p=0.293	0.19 (-0.15, 0.53) p=0.269	0.25 (-0.10, 0.60) p=0.166
**2-4 CMBs**	0.23 (-0.31, 0.76) p=0.403	0.38 (-0.16, 0.92) p=0.164	0.01 (-0.53, 0.55) p=0.967	0.19 (-0.15, 0.53) p=0.269	0.19 (-0.35, 0.73) p=0.487
**>4 CMBs**	1.06 (-0.61, 2.72) p=0.214	-0.83 (-2.51, 0.85) p=0.335^#^	**2.14 (0.45, 3.84) p=0.013**	**1.81 (0.18, 3.45) p=0.030**	4.30 (2.62, 5.97) p<0.001*^#^
**Mixed-location CMBs (counts)**	0.00 (-0.00, 0.01) p=0.303	0.00 (-0.00, 0.01) p=0.373	-0.00 (-0.01 0.01) p=0.958^#^	0.01 (-0.01, 0.01) p=0.112	0.00 (-0.03, 0.01) p=0.437
**2-4 CMBs**	0.19 (-0.17, 0.56) p=0.296	0.18 (-0.19, 0.55) p=0.340	0.19 (-0.18, 0.57) p=0.306	0.23 (-0.13, 0.59) p=0.205	0.08 (-0.29, 0.46) p=0.664
**>4 CMBs**	0.04 (-0.26, 0.33) p=0.806^#^	-0.04 (-0.34, 0.25) p=0.787^#^	-0.05 (-0.35, 0.25) p=0.727^#^	0.16 (-0.13, 0.44) p=0.287	0.06 (-0.24, 0.36) p=0.701

All values adjusted for age, gender, intracranial volume, hypertension, hyperlipidemia and diabetes mellitus.

Bold values represents statistically significant associations at p<0.05.

* Statistically significant after Bonferroni correction (0.05/4 ~ 0.013).

^#^ Statistically significant after further adjusting for white matter hyperintensities volume, presence of lacunes and total enlarged perivascular spaces (n=328) (p<0.05).

### Subgroup analysis with other MRI markers

In the subgroup analysis consisting of 328 patients, mixed-location CMBs were associated with smaller global cortical thickness [β= -0.03; 95% CI= -0.05, -0.01, p=0.013] as well as cortical thickness in frontal [β= -0.02; 95% CI= -0.04, -0.00, p=0.043], parietal [β= -0.02; 95% CI= -0.04, -0.00, p=0.004], occipital [β= -0.02; 95% CI= -0.04, -0.00, p=0.020] and limbic [β= -0.03; 95% CI= -0.05, -0.01, p=0.010] lobes, smaller brainstem volume [β= -0.03; 95% CI= -0.04, -0.01, p=0.001] and smaller volumes of all the hippocampus subfields (p<0.05) when further adjusted for total WMH volumes, presence of lacunes and total ePVS. Similarly, strictly lobar CMBs were associated with smaller total white matter volume and region specific white matter volumes in frontal, parietal, temporal and occipital lobes (p<0.05) [data not shown].

### Stratified analysis by diagnostic groups

On performing secondary analysis among patients with NCI, CIND and dementia, we found that in patients with CIND, strictly deep CMBs were associated with smaller cortical thickness in temporal lobe [β= -0.14; 95% CI= -0.27, -0.01, p=0.040] and with larger parietal [β= 0.16; 95% CI= 0.02, 0.31, p=0.027], temporal [β= 0.14; 95% CI= 0.00, 0.27, p=0.044] and occipital [β= 0.33; 95% CI= 0.20, 0.47, p<0.001] white matter volumes whereas, mixed-location CMBs were associated with smaller accumbens volume [β= -0.03; 95% CI=- 0.04, -0.01, p=0.001]. However, in patients with dementia, mixed-location CMBs were associated with larger thalamic [β= 0.02; 95% CI= 0.02, 0.03, p<0.001] and lentiform nucleus volumes [β= 0.01; 95% CI= 0.00, 0.02, p=0.010] and smaller total and region specific white matter volumes (p<0.05). No significant associations were found between strictly lobar, strictly deep and mixed-location CMBs with hippocampus subfield volumes among patients with NCI, CIND and dementia [data not shown].

## DISCUSSION

The present study demonstrated that mixed-location CMBs were associated with cortical thinning in frontal, temporal, and limbic lobes and smaller accumbens volume independent of demographics, vascular risk factors and intracranial volume. These associations were more specifically observed in patients who had more than 4 mixed-location CMBs. Mixed-location CMBs were also associated with smaller hippocampus subfield volumes, more specifically in hippocampal tail, subiculum, presubiculum, molecular, GCMLDG, CA3, and CA4. Subjects with strictly lobar CMBs had smaller total and region specific white matter volumes. Mixed-location CMBs were associated with larger thalamic volume and strictly deep CMBs were associated with larger parietal, temporal and occipital white matter volumes.

Previous studies have suggested that CMBs might be a consequence of two separate pathways that act as a catalyst towards subsequent neurodegeneration [20]. Although in patients with intracranial hemorrhage, it has been shown that CAA and hypertensive arteriopathy play a role in the pathophysiology of CMBs and dementia [3, 21]. The present study adds further to the previous findings by demonstrating that cerebral atrophy may be a possible consequence of mixed pathology. Previous histopathological studies have shown controversial results where one study reported that cerebral amyloid is typically deposited in the cortical vessels and manifests as lobar CMBs [22], whereas another study has shown the presence of lobar CMBs in patients with hypertensive arteriopathy [19]. When similar concepts were extrapolated in observation studies, it was reported that the hypertensive arteriopathy may also cause lobar CMBs [17, 18, 23, 24]. It is further shown that 50% of CAA patients have hypertension suggesting underlying mixed pathology in CAA [25]. Our current findings of an association between mixed-location CMBs with neurodegenerative markers may be due to presence of both CAA and hypertensive arteriopathy. Amyloid-beta deposited in blood vessels not only leads to the destruction of vessel wall and development of CMBs, but may also give rise to ischemic manifestations [26]. Moreover, hypertensive arteriopathy due to lipohyalinosis and arteriosclerosis of cerebral blood vessels may also compromise cerebral blood flow [27–29]. Apart from vascular amyloid, parenchymal amyloid deposition may also lead to neuronal loss [30]. Hence, cortical atrophy in the presence of mixed-location CMBs may also be due to secondary axonal or trans- synaptic degeneration [31]. Fronto-subcortical circuits which connect frontal lobe with subcortical structure may be disrupted due to presence of CMBs in deep region. Thus, atrophy of frontal lobe and other brain regions might be expected due to mixed-location CMBs-associated secondary degeneration [31].

With regards to subcortical structures, we found that mixed-location CMBs were associated with smaller volumes of accumbens and hippocampal subfields. . These associations may be explained by the fact that these subcortical structures are interconnected by neural circuits [32] and are particularly vulnerable to deposition of amyloid protein [33, 34]. It has been shown that intraneuronal aggregates of tau, first target the entorhinal cortex and spread into hippocampus, causing damage to the neuronal networks in the hippocampus [35, 36]. Furthermore, it has been shown that CA3 atrophy is associated with amyloid β [37]. Thus, subcortical atrophy in these regions may be due to a disruption in neuronal circuits connection or secondary degeneration due to deposition of amyloid-beta aggregates which triggers myelin loss and degeneration of connecting neurons [38]. Furthermore, cerebral hypoperfusion due to mixed pathology may be the cause of subcortical atrophy. In age, gender, vascular risk factors and other MRI markers of SVD adjusted models, we found mixed-location CMBs were associated with smaller brain stem volume. It has been shown that deposition of neurofibrillary tangles and alterations of neurotransmitters in brain stem may occur in patients with Alzheimer’s disease [39]. In this study, mixed-location CMBs were also associated with larger thalamic volume and the presence of >4 mixed-location CMBs were associated with larger lentiform nucleus volume. These associations were mostly seen in patients with dementia. There could be two possible explanations to these findings; firstly, it has been reported that increased neuronal hypertrophy and/or inflammation are typically observed in AD pathology giving rise to increase volumes of lentiform and thalamus [39]. Secondly, periventricular white matter hyperintensities are difficult to distinguish from gray matter especially the caudate nucleus on T1 sequences and hence may lead to an artificially increased volume of basal ganglia nuclei [39]. Interestingly, in our analyses, we observed that the associations of mixed microbleeds with neurodegenerative markers were specifically observed in patients without dementia suggesting that neurodegenerative process starts early in the preclinical stages of dementia but eventually plateaus in late stages of dementia.

We also found that strictly lobar CMBs were associated with smaller total and region specific white matter volumes. Previously it has been reported that CMBs in cortical region of the brain may cause axonal degeneration triggering myelin loss and ultimately leading to dysfunction and degeneration of connecting axons in white matter [38]. Furthermore, deposition of amyloid-beta in leptomeningeal vessels may cause hypoperfusion leading to ischemic damage and white matter atrophy in patients with dementia [26, 29, 40]. Our findings are thus in line with the previous findings. Interestingly, we found that deep CMBs, more specifically in patients with CIND were associated with larger parietal, temporal and occipital white matter volume. This may be explained by the fact that, CMBs which consist of hemosiderin and other extravagated blood products in the brain may cause blood brain barrier dysfunction and inflammatory changes in brain tissue leading to edema of neurons [41]. Another explanation in the current literature revolves around the possibility that amyloid deposition in the blood vessels may cause insufficient drainage of interstitial fluids increasing its accumulation in brain tissue [42]. Previous study has also shown increased white matter free water content in the patients with cerebrovascular disease [43].

Though, it has been consistently shown that CAA is the major pathological mechanism for lobar CMB and hypertensive arteriopathy for deep CMBs [1]; however, our data did not support these findings. This may be due to the presence of mixed pathology in our population. We postulate that CAA and hypertensive arteriopathy might be two separate mechanisms in preclinical stages, but at the advanced stage they co-occur and manifest as mixed pathology [40]. Hence, it is also possible that cerebral atrophy in this study may be due to pre-existing neurodegenerative pathology or CAA/ hypertensive arteriopathy-related microvascular ischemic changes [12].

Limitations of this study include; first, as most of our study subjects were recruited from memory clinic with different risk factors profile, our findings may not be generalizable to the general population. Second, even though we adjusted for several risk factors and MRI markers of SVD, we cannot ignore the possibility of residual confounding. Third, we were unable to determine whether cerebral atrophy in our patients was due to CMBs-related microvascular changes or due to pre-existing neurodegeneration. Nevertheless, intracranial volume was treated as an important confounder in all our models. Fourth, we were unable to explore the temporal relationship between mixed-location CMBs and neurodegeneration due to cross-sectional design. Fifth, even though we have included 477 participants in this study, the sample size for CMBs in various groups (strictly lobar= 107, strictly deep =35 and mixed-location=58) were relatively small which may underestimate effect sizes. Finally, lobar CMBs detected on MRI may provide an indirect measure of CAA or amyloid deposition and deep CMBs may provide an indirect measurement of atherosclerosis and lipohyalinosis in cerebral blood vessels. Due to lack of positron emission tomography images and pathological data, we were unable to confirm our findings. Hence, our results should be interpreted with caution. Strengths of this study include, automated and standardized image processing to quantify neurodegenerative markers. CMBs were graded blinded to clinical findings which prevented overestimation of CMBs.

## CONCLUSIONS

The present study showed that mixed-location CMBs were independently associated with cortical and subcortical atrophy whereas strictly lobar CMBs were associated with white matter atrophy. Our findings suggest a shared mechanism of vascular dysfunction due to deposition of amyloid beta, lipohyalinosis and arteriosclerosis of cerebral blood vessels. Thus, greater emphasis should be made on treating vascular risk factors so as to prevent CMBs-related neurodegeneration and cognitive changes.

## MATERIALS AND METHODS

### Study population

For this study, data was obtained from the ongoing prospective memory clinic based case-control study. The controls were individuals who may have subjective cognitive complains, but no objective cognitive impairment on comprehensive neuropsychological tests or functional impairment and were diagnosed as No Cognitive Impairment (NCI). Cases were participants with subjective memory complains and impairment on neuropsychological assessment and were diagnosed with cognitive impairment no dementia (CIND) and dementia. CIND was defined as impairment in at least one cognitive domain on comprehensive neuropsychological test, but did not meet the criteria for dementia according to the Diagnostic and Statistical Manual for Mental Disorder-Fourth Edition (DSM-IV). Dementia was diagnosed according to the DSM- IV criteria. The etiological diagnoses of dementia were based on internationally accepted criteria: Alzheimer’s Disease (AD) was diagnosed using the National Institute of Neurological and Communicative Disorders and Stroke and the Alzheimer's Disease and Related Disorders Association (NINCDS-ADRDA), AD with cerebrovascular disease or mixed dementia was defined as subjects fulfilling criteria of diagnosis of AD together with concomitant cerebrovascular disease on MRI (presence of multiple infarcts or extensive white matter hyperintensities) and vascular dementia (VaD) was defined using the National Institute of Neurological Disorders and Stroke and Association Internationale pour la Recherché et l' Enseignement en Neurosciences (NINDS-AIREN) criteria [44–46].

A total of 578 patients were recruited for this study from August 2010 to January 2016. All subjects underwent comprehensive evaluation including physical, medical and neuropsychological assessment along with 3T magnetic resonance imaging (MRI), all done on the same day. Of 578 patients, 12 did not perform MRI scans (3 were claustrophobic, 1 refused MRI, 2 were uncooperative and could not follow instructions properly and 6 could not perform MRI due to other medical conditions), 18 patients with incomplete and poor quality MRI scan and 71 patients with cortical stroke were excluded, leaving 477 patients for the final analysis [NCI=104 (21.8%), CIND=211 (44.2%) and Dementia=162 (34.0%) (AD=65; 40.1%, VaD=20; 12.3%, mixed dementia=77; 47.6%)].

This study was approved by the National Healthcare Group Domain-Specific Review Board and was conducted in accordance with the Declaration of Helsinki. A written informed consent was obtained from all subjects or their caregivers prior to the recruitment for this study.

### Covariates

A detailed demographic questionnaire was administered for each subject to collect information on age, gender, years of formal education and smoking. History of hypertension, hyperlipidemia, and diabetes mellitus was noted and verified with medical records. Hypertension was defined as systolic blood pressure ≥140mmHg and /or diastolic blood pressure ≥90mmHg or a history of hypertension, or use of antihypertensive medication. Hyperlipidemia was defined as total cholesterol level ≥4.14 mmol/l or a history of hyperlipidemia, or use of lipid-lowering medication. Diabetes mellitus was defined as glycated hemoglobin ≥6.5% or a history of diabetes mellitus, or the use of any glucose-lowering medication.

### Neuroimaging

MRI scans of all the patients were performed at the Clinical Imaging Research Center of the National University of Singapore, using 3T Siemens Magnetom Trio Tim Scanner system, with a 32-channel head coil. The standardized neuroimaging protocol in this study included a three dimensional T1-weighted, T2-weighted, fluid-attenuated inversion recovery (FLAIR) and susceptibility weighted image (SWI) sequences. SWI sequence was used to detect CMBs with echo time = 20 ms; repetition time= 27 ms; flip angle= 15 degrees; field of view= 256 mm; field of view= 75%; image matrix= 192x256; slice thickness= 1.50 mm.

### Cerebrovascular disease markers

MRI markers of cerebrovascular disease (lacunes, white matter hyperintensities, enlarged perivascular spaces and cerebral microbleeds) were graded using the Standards for Reporting Vascular changes on Neuroimaging (STRIVE) criteria [47].

1. CMBs were graded according to the Microbleed Anatomical Rating Scale [48]. CMBs were classified manually into two different locations: lobar (frontal, parietal, temporal, occipital and insula) and deep (basal ganglia, thalamus, internal and external capsule, brainstem and cerebellum). Based on the location of CMBs, patients were classified into 3 groups: strictly lobar CMBs (having CMBs exclusively in lobar region), strictly deep CMBs (having CMBs exclusively in deep region) and mixed-location CMBs (having CMBs in both lobar and deep locations). The total number of CMBs in each location was recorded. Both strictly lobar and strictly deep CMBs were further categorized in three groups according to CMBs burden: presence of 1 CMB, presence of 2-4 CMBs and presence of >4 CMBs, whereas mixed-location CMBs were categorized into two groups: presence of 2-4 CMBs and presence of >4 CMBs [49, 50].
2. Lacunes were defined as round or ovoid lesions, 3 to 15 mm in diameter in the subcortical regions, with a high signal on T2-weighted images and a low signal on T1-weighted and FLAIR images, and a hyperintense rim with a center following the cerebrospinal fluid intensity [47].
3. WMH volume was quantified using T1 and T2 weighted images. Image preprocessing and the tissue classification algorithm have been described elsewhere [51]. Region-specific WMH volume (ml) was calculated for frontal, parietal, occipital and temporal lobes. Total WMH volume was calculated as the sum of WMH volumes in above mentioned 5 regions [52].
4. ePVS were defined as round or linear lesion, which were hypointense on T1 weighted and hyperintense on T2 weighted images. It was considered dilated when lesion was ≥1mm in diameter. ePVs were visually graded in 4 different regions of brain: centrum semiovale, basal ganglia, mesencephalon and hippocampus. Total numbers of ePVS in each region were recorded. Total ePVS was calculated as sum of centrum semivale, basal ganglia, hippocampus and mesencephalon ePVS [52].

All the MRI scans were graded by two independent raters (B.G & M.A.S) blinded to the clinical history. All potential MRI markers of SVD were discussed in the weekly consensus meeting. Any disagreement was further discussed with an experienced neuroimaging fellow (SH) to make a final decision. Inter-rater and intra-rater agreement was excellent, which has been published previously [53, 54].

### Neurodegenerative markers

Quantitative MRI analyses were performed using automated segmentation procedure at the Department of Medical Informatics, Erasmus University Medical Center, Netherlands using a model based automated procedure (FreeSurfer, v.5.1.0) on T1 weighted images (TR=7.2 ms, TE=3.3 ms, matrix=256×256×180 mm^3^). For each patient, the following MRI markers were computed.

1. Cortical thickness was measured as the shortest distance between gray/white matter boundary and pial surface at each vertex. Global and regional thickness averages were converted to millimeters (mm). Regional average was calculated from left and right lobes thickness using parcellation guide on sulcus and gyrus of cerebral cortex. Region specific cortical thicknesses were calculated for the frontal, parietal, occipital, temporal, insular, and limbic regions [55].
2. Subcortical structures volumes were calculated for accumbens, amygdala, lentiform nucleus, thalamus, hippocampus, and brainstem. Segmentation was performed using rigid body registration and nonlinear normalization of images to a probabilistic brain atlas. During segmentation process, each voxel was labeled automatically as corresponding brain region on parcellation guide. Volumes of accumbens, amygdala, lentiform nucleus, hippocampus and thalamus and were calculated for left and right hemispheres separately. Finally, average volumes of each structure were calculated and then converted into milliliters (ml) [39, 56].
3. Hippocampus subfield volumes were calculated in 12 subfields based on an ultra-high resolution ex-vivo atlas and includes: Cornu Amonis regions 1, 2 and 3 combined, and 4 (CA1, CA3, and CA4), parasubiculum, presubiculum, subiculum, molecular and granule cell layer of dentate gyrus (GCMLDG), hippocampus amygdala transition area (HATA), fimbria, molecular layer, hippocampal fissure, and hippocampal tail. For each subject both left and right hemisphere hippocampus subfield volumes were calculated separately. Finally, average volumes of each subfield were calculated and then converted into ml.
4. White matter volume was quantified using T1 and T2 weighted images. The used image preprocessing steps and the tissue classification algorithm have been described elsewhere [51]. Briefly, k-nearest-neighbor brain tissue classifier technique was used to classify voxels into cerebrospinal fluid (CSF), gray matter, white matter, white matter hyperintensities (WMH). Volume (ml) was calculated for all biomarkers from these segmentations. Region-specific white matter volume was calculated for frontal, parietal, occipital and temporal lobes. Total white matter volume was calculated as the sum of white matter volumes in the above mentioned five regions. Intracranial volume was the sum of the CSF, gray matter, white matter and WMH.

### Statistical analysis

In order to examine the differences in the baseline characteristics of patients with and without CMBs, t-test was performed for normally distributed continuous variables (age, total WMH volume and neurodegenerative markers), Mann-Whitney U Test for skewedly distributed continuous variable (total ePVs), and chi-square test for binary variables (gender, hypertension, hyperlipidemia, diabetes and presence of lacunes). For regression analysis logarithmical transformation of WMH volumes was performed to ensure normal distribution. Other neurodegenerative markers: global cortical thickness, region specific cortical thickness, total white matter volume, region specific white matter volumes, subcortical structures volumes and hippocampus subfields volumes were standardized by dividing each variable by its standard deviation [39, 55]. CMBs were treated as both count and categorical data. For categorical data, we classify CMBs by location: presence of strictly lobar vs absence, presence of strictly deep vs absence and presence of mixed-location vs absence and by numbers: strictly lobar and strictly deep CMBs as 1 vs absence, 2-4 vs absence and >4 vs absence and mixed-location CMBs as 2-4 vs absence, and >4 vs absence. Total numbers of CMBs in each location: strictly lobar, strictly deep and mixed-location were treated as count data. Linear regression models were performed to determine the association between CMBs with cortical thickness (global and region specific thickness), white matter volumes (total and region specific volumes), subcortical structure volumes (accumbens, amygdala, lentiform nucleus, thalamus and brainstem) as well as hippocampus subfield volumes (CA1, CA3, CA4, parasubiculum, presubiculum, subiculum, GCMLDG, HATA, fimbria, molecular layer, hippocampal fissure, and hippocampal tail). In our stratified analysis, we divided our patients into three groups i.e. patients with NCI, CIND and dementia (AD, VaD and mixed dementia). In the analysis we treated CMBs as determinants and cortical thickness, subcortical structures volume and white matter volume as outcomes. Regression models were first adjusted for age, gender, intracranial volume, hypertension, hyperlipidemia, and diabetes. Furthermore, in a subgroup of 328 patients regression models were further adjusted for presence of lacune, total ePVs and total WMH volume (ePVS grading were only available for 328 patients). Mean difference with 95% confidence intervals (CIs) from the regression models were reported.

Results were considered significant at p<0.05. In view of multiple testing performed between CMBs and neurodegenerative markers, we used Bonferroni correction to obtain revised statistical significance level of 0.05/6 ~0.0083 for cortical thickness and 0.05/5~0.010 for subcortical structural volumes. When analyzing the association between CMBs and region specific white matter volume, we used the revised statistical significant level set at 0.05/4 ~ 0.013. Similarly, when analyzing association between CMBs and hippocampus subfield volumes statistical significance level was set at 0.05/12~0.0041. All data were analyzed using SPSS software package (version 25).

## Supplementary Material

Supplementary Tables
