# Supplementary material for: Mixed-location cerebral microbleeds as a biomarker of neurodegeneration in a memory clinic population
Source: Aging (Albany NY). 2019 Nov 25;11(22):10581–96. doi: 10.18632/aging.102478 (PMC6914397; doi:10.18632/aging.102478)
Supplement: Supplementary Tables [file aging-11-102478-s001..pdf]

SUPPLEMENTARY TABLES

Supplementary Table 1. Association between CMBs and cortical thickness

	Global cortical thickness mean difference (95%CI)	Region specific cortical thickness, mean difference (95%CI)					
		Frontal	Parietal	Temporal	Occipital	Limbic	Insula
Presence of strictly lobar CMBs	-0.04 (-0.25, 0.17) p=0.705	0.08 (-0.14, 0.30) p=0.473	-0.12 (-0.33, 0.09) p=0.261	-0.06 (-0.27, 0.15) p=0.597	-0.09 (-0.03, 0.12) p=0.406	0.03 (-0.19, 0.24) p=0.813	0.15 (-0.07, 0.37) p=0.175
Presence of strictly deep CMBs	0.01 (-0.33, 0.35) p=0.974	-0.02 (-0.37, 0.33) p=0.910	0.04 (-0.30, 0.38) p=0.807	0.07 (-0.26, 0.41) p=0.678	-0.14 (-0.47, 0.19) p=0.397	-0.04 (-0.38, 0.30) p=0.808	0.04 (-0.31, 0.39) p=0.808
Presence of mixed-location CMBs	-0.26 (-0.53, 0.02) p=0.064	**-0.29 (-0.57, -0.01) p=0.042**	-0.09 (-0.37, 0.18) p=0.512	**-0.29 (-0.56, -0.02) p=0.035**	-0.16 (-0.43, 0.11) p=0.245	**-0.35 (-0.63, -0.08) p=0.011**	-0.23 (-0.51, 0.05) p=0.107

All values adjusted for age, gender, intracranial volume, hypertension, hyperlipidemia and diabetes
Bold values represent statistically significant associations at p =0.05
* Statistically significant after Bonferroni correction (0.05/6 ~ 0.0083)
# Statistically significant after further adjusting for white matter hyperintensities volume, presence of lacunes and total enlarged perivascular spaces (n=328) (p<0.05)

Supplementary Table 2. Association between CMBs and subcortical structures volume.

	Accumbens Mean difference (95%CI)	Amygdala Mean difference (95%CI)	Lentiform Mean difference (95%CI)	Thalamus Mean difference (95%CI)	Hippocampus Mean difference (95%CI)	Brainstem Mean difference (95%CI)
Presence of strictly lobar CMBs	-0.04 (-0.24, 0.16) p=0.175	-0.16 (-0.35, 0.03) p=0.105	**-0.32 (-0.52, 0.12) p=0.002***	-0.08 (-0.26, 0.10) p=0.385	-0.21 (-0.40, -0.02) p=0.034	-0.04 (-0.22, 0.14) p=0.683
Presence of strictly deep CMBs	-0.03 (-0.35, 0.29) p=0.848	0.10 (-0.20, 0.41) p=0.500	0.08 (-0.24, 0.41) p=0.622	0.09 (-0.20, 0.38) p=0.542	0.11 (-0.20, 0.42) p=0.483	-0.16 (-0.45, 0.13) p=0.272
Presence of mixed-location CMBs	**-0.30 (-0.56, -0.04) p=0.024**	-0.08 (-0.33, 0.16) p=0.511	**0.41 (0.15, 0.67) p=0.002***	-0.22 (-0.45, 0.02) 0.068#	-0.05 (-0.30, 0.20) p=0.702	**-0.35 (-0.58, -0.12) p=0.003*#**

All values adjusted for age, gender, intracranial volume, hypertension, hyperlipidemia and diabetes mellitus
Bold values represent statistically significant associations at p =0.05
* Significant after Bonferroni correction (0.05/5 ~0.010)
# Statistically significant after further adjusting for white matter hyperintensities volume, presence of lacunes and total enlarged perivascular spaces (n=328) (p<0.05)

Supplementary Table 3. Association between CMBs and white matter volume.

	Total white matter volume (ml) mean difference (95%CI)	Lobe specific white matter volume (ml), mean difference (95%CI)			
		Frontal	Parietal	Temporal	Occipital
Presence of strictly lobar CMBs	-0.18 (-0.36, 0.01) p=0.060	-0.16 (-0.35, 0.03) p=0.096	-0.16 (-0.34, 0.03) p=0.107	**-0.20 (-0.38, 0.02) p=0.030**	-0.16 (-0.35, 0.04) p=0.109
Presence of strictly deep CMBs	**0.26 (-0.03, 0.56) p=0.0.081**	0.20 (-0.10, 0.49) p=0.198	0.19 (-0.11, 0.50) p=0.209	0.29 (-0.01, 0.58) p=0.055	**0.34 (0.04, 0.65) p=0.027**
Presence of mixed-location CMBs	0.10 (-0.14, 0.34) p=0.422	0.04 (-0.20, 0.28) p=0.721	0.04 (-0.20, 0.28) p=0.736	0.19 (-0.05, 0.42) p=0.121	0.07 (-0.18, 0.31) p=0.584

All values adjusted for age, gender, intracranial volume, hypertension, hyperlipidemia and diabetes mellitus
Bold values represent statistically significant associations at p =0.05
* Statistically significant after Bonferroni correction (0.05/4 ~ 0.013)
# Statistically significant after further adjusting for white matter hyperintensities volume, presence of lacunes and total enlarged perivascular spaces (n=328) (p<0.05)
